# Supplementary figures and images for: Combined analyses of RNA-sequence and Hi-C along with GWAS loci—A novel approach to dissect keloid disorder genetic mechanism
Source: PLoS Genet. 2022 Jun 16;18(6):e1010168. doi: 10.1371/journal.pgen.1010168 (PMC9202908; doi:10.1371/journal.pgen.1010168)

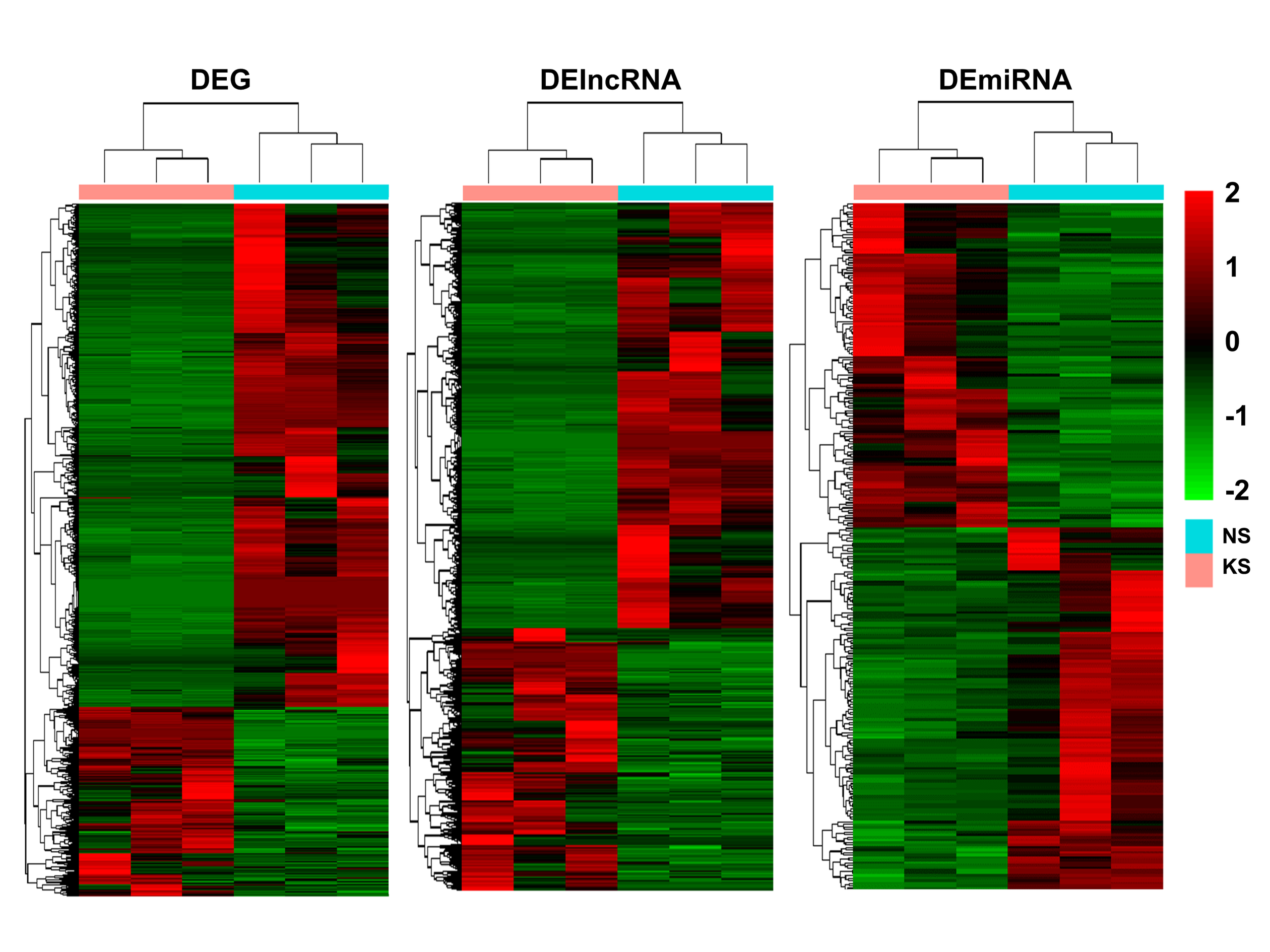

Supplement: S1 Fig — Heatmap showed DEGs, DElncRNAs and DEmiRNAs between keloids and their adjacent normal skins. (TIF) [file pgen.1010168.s001.tif]

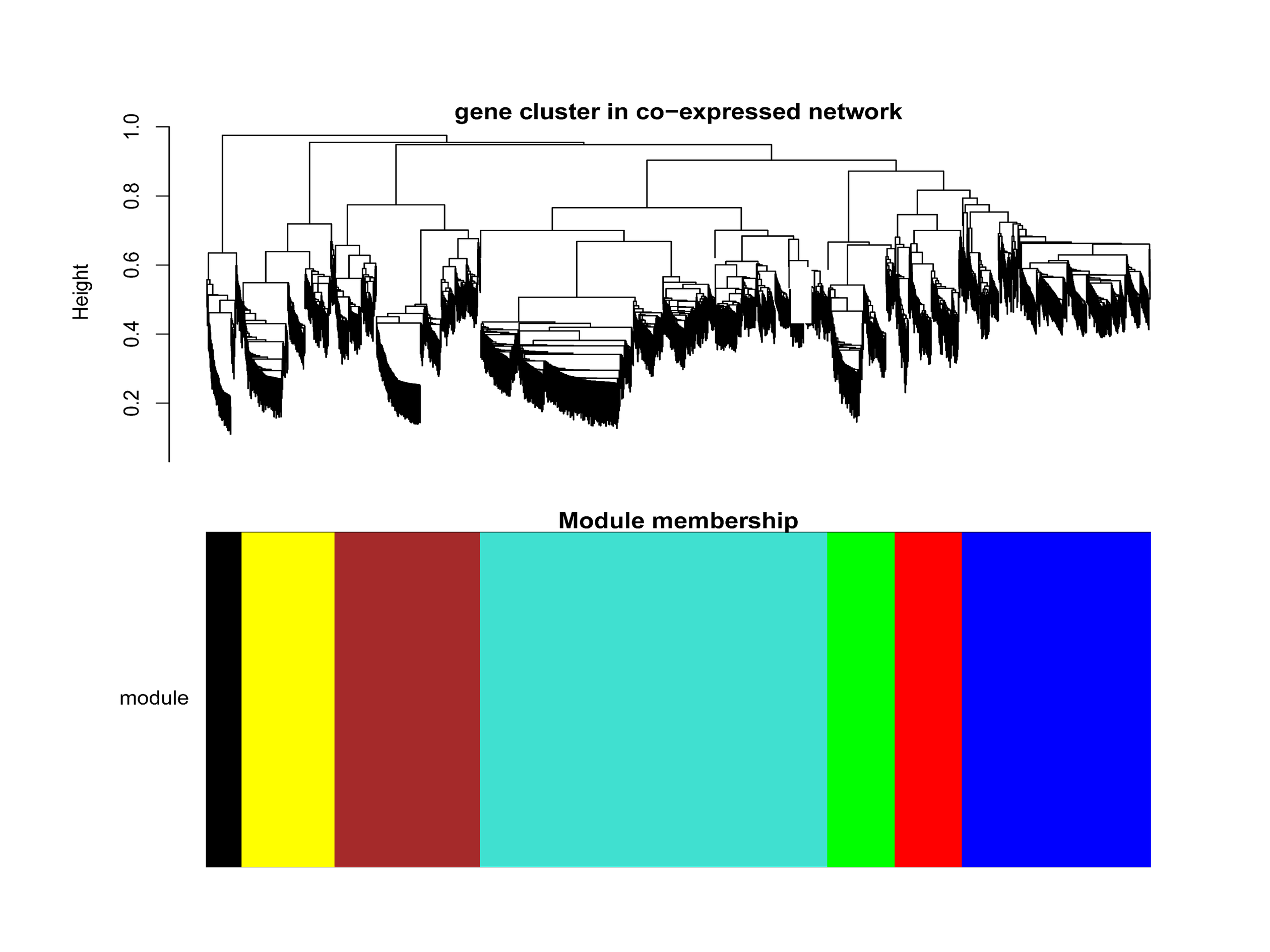

Supplement: S2 Fig — (TIF) [file pgen.1010168.s002.tif]

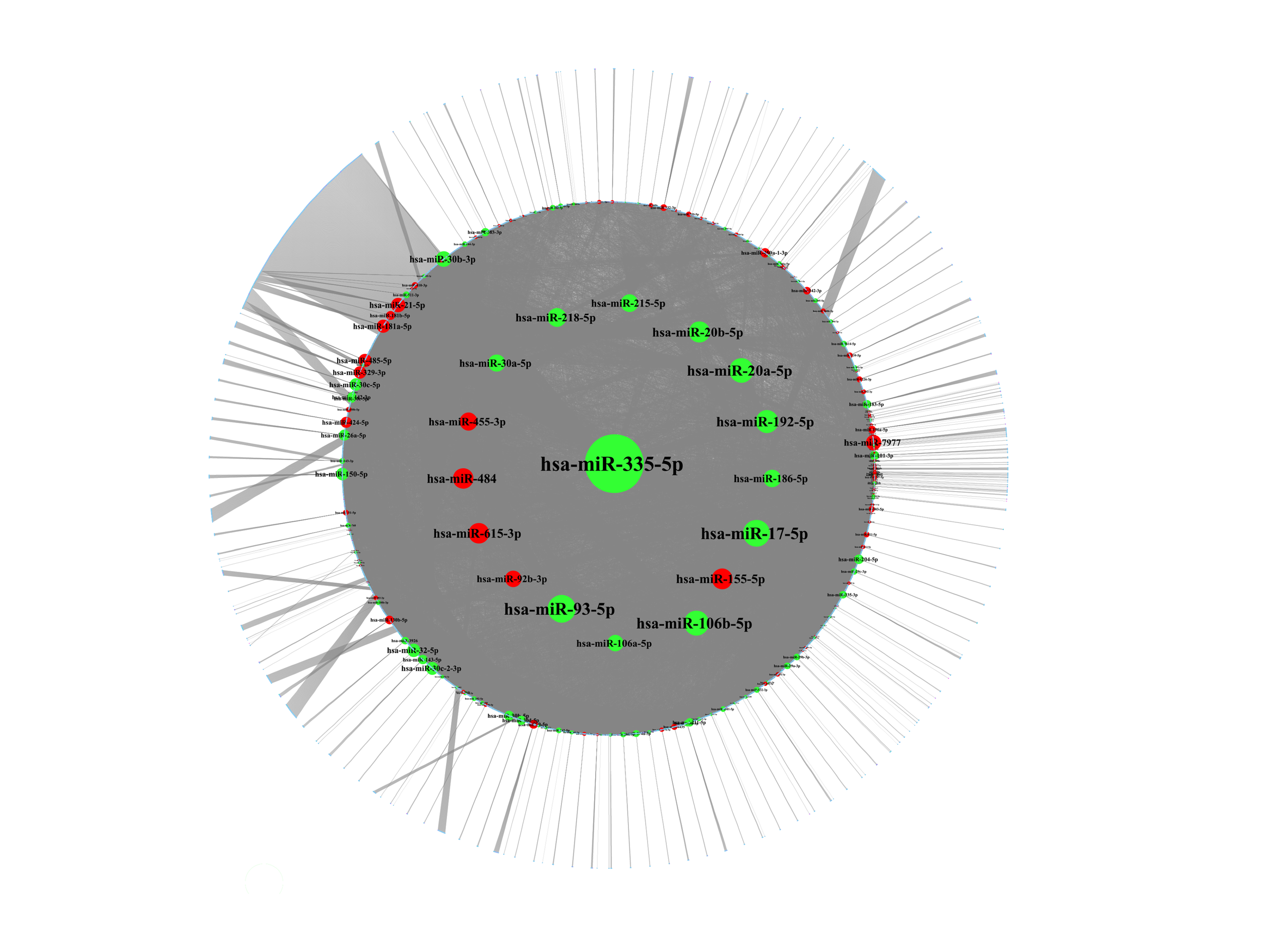

Supplement: S3 Fig — (TIF) [file pgen.1010168.s003.tif]

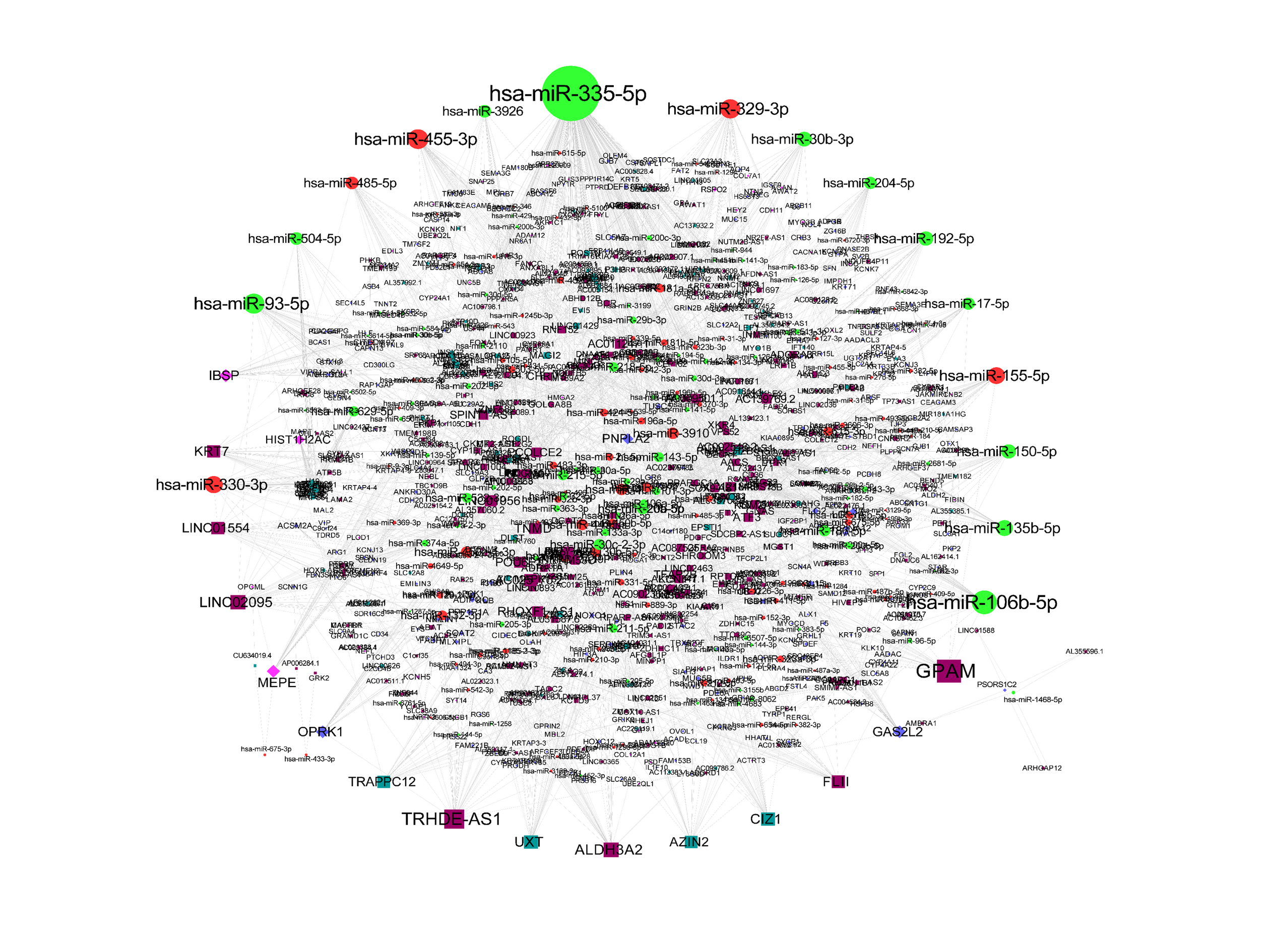

Supplement: S4 Fig — (TIF) [file pgen.1010168.s004.tif]

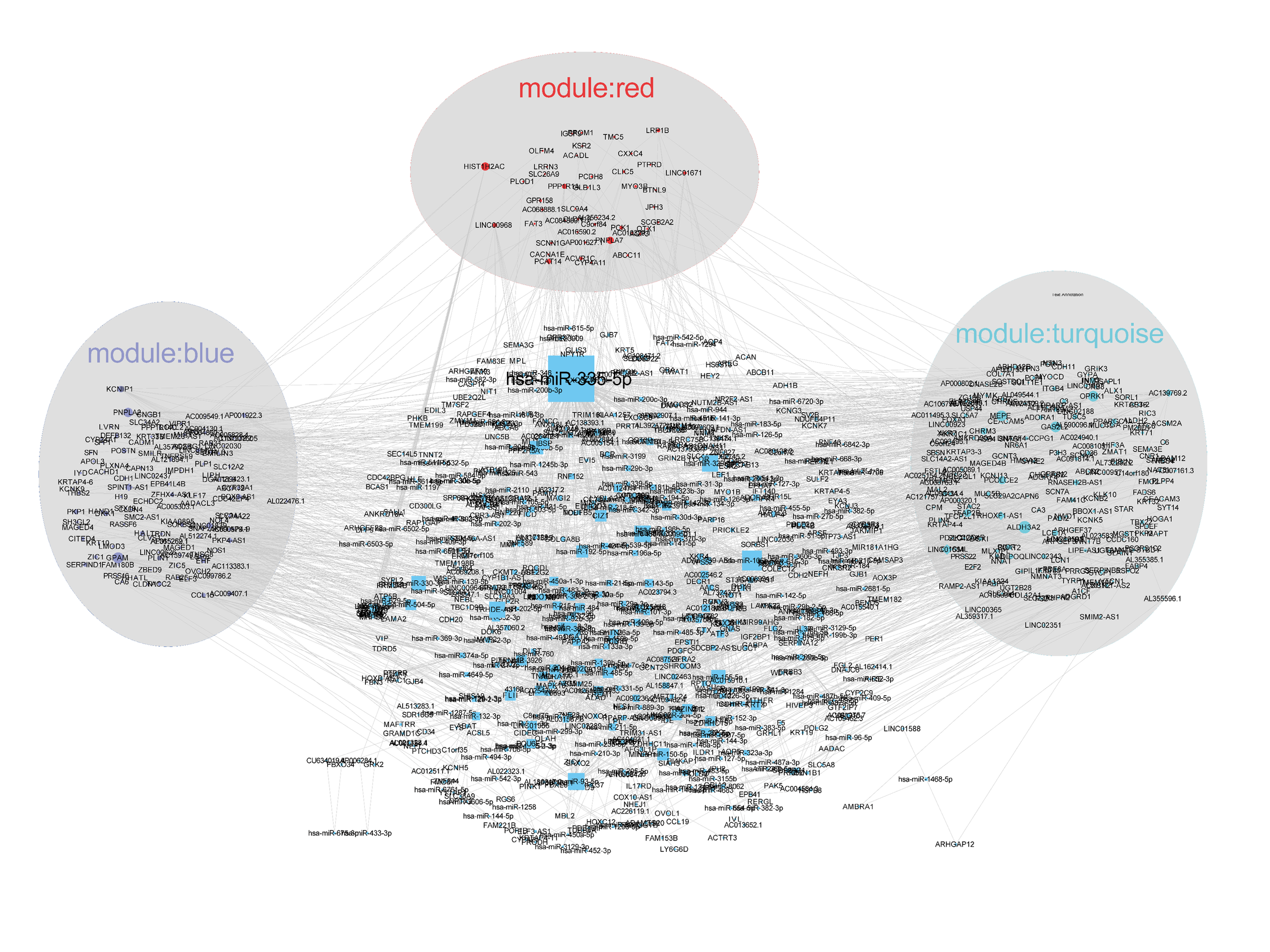

Supplement: S5 Fig — (TIF) [file pgen.1010168.s005.tif]

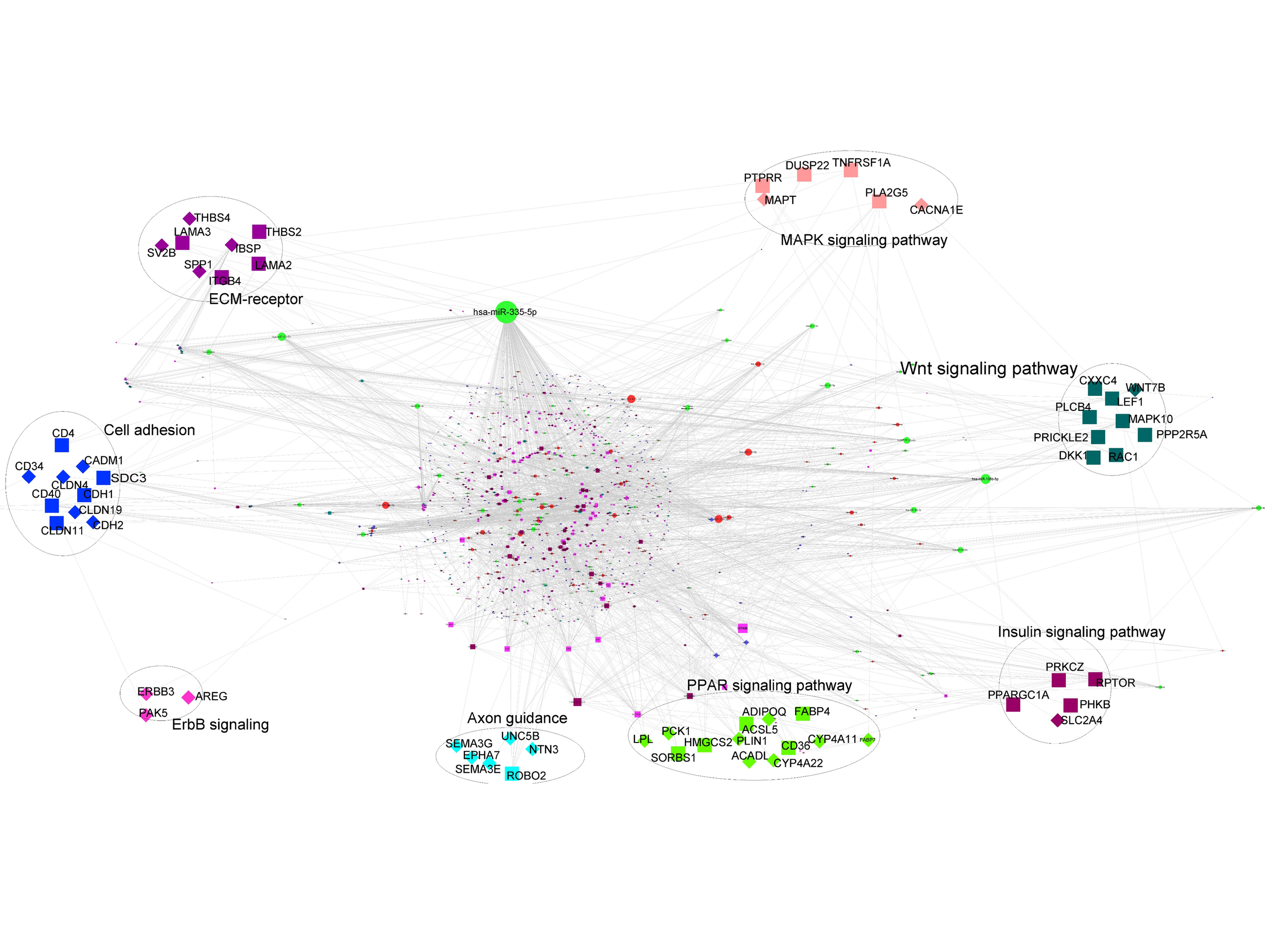

Supplement: S6 Fig — (TIF) [file pgen.1010168.s006.tif]

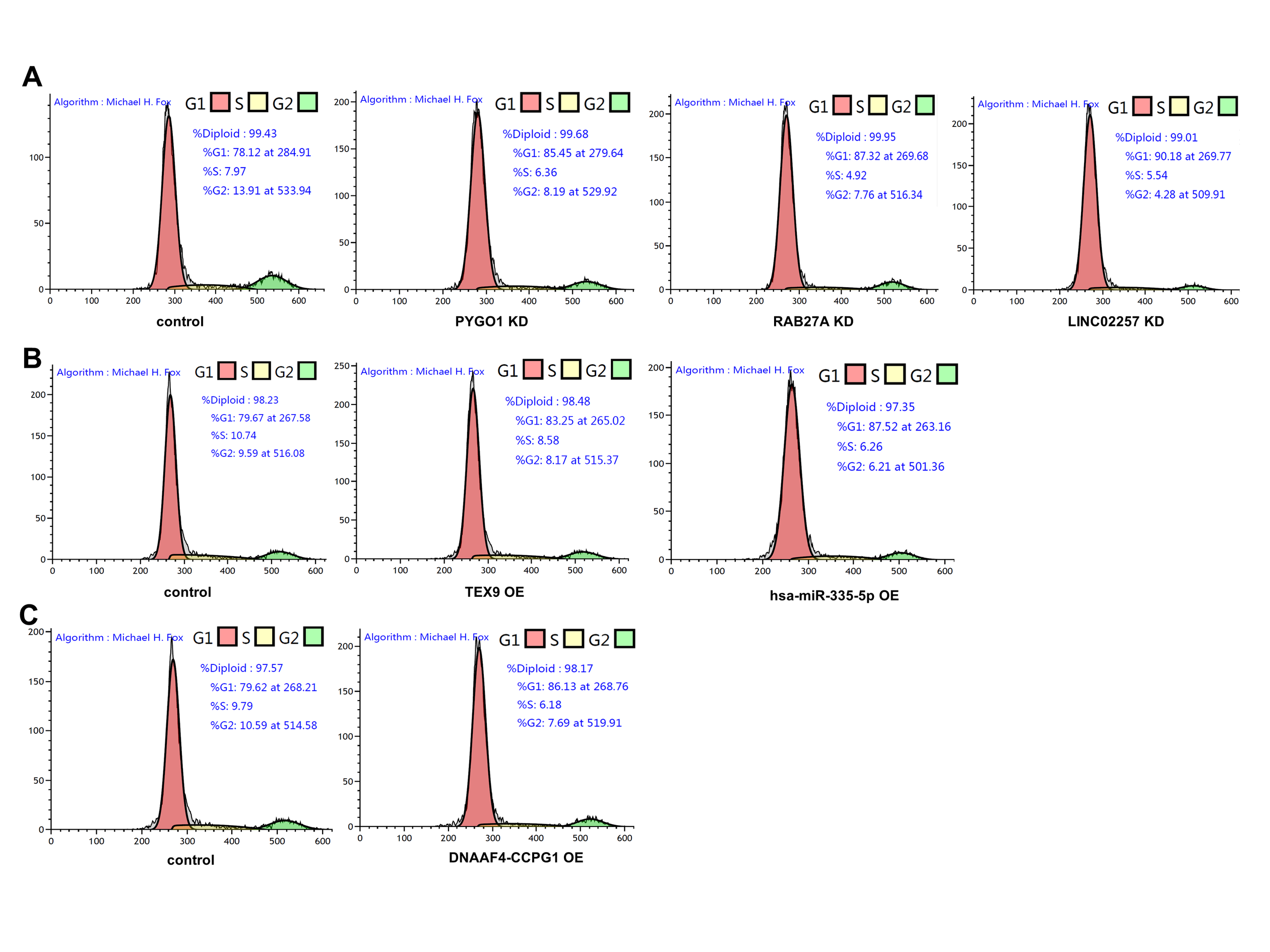

Supplement: S7 Fig — Representative histograms of cell cycle phase distribution of KFs with knockdown of PYGO1, RAB27A, LINC02257 (A) and overexpression of TEX9, hsa-miR-335-5p (B) and DNAAF4-CCPG1 (C). (TIF) [file pgen.1010168.s007.tif]

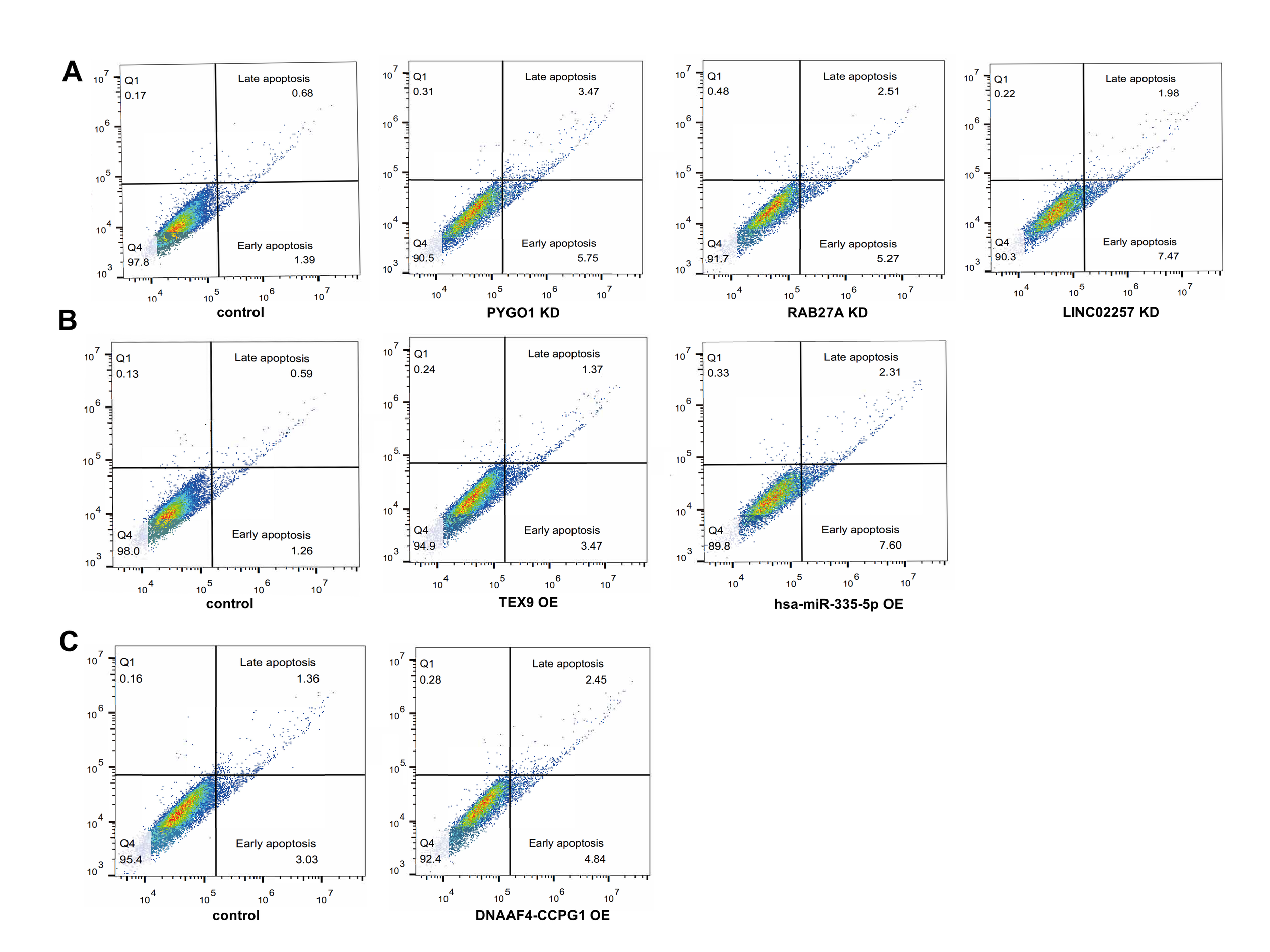

Supplement: S8 Fig — Representative apoptosis pattern of KFs with knockdown of PYGO1, RAB27A, LINC02257 (A) and overexpression of TEX9, hsa-miR-335-5p (B) and DNAAF4-CCPG1 (C). (TIF) [file pgen.1010168.s008.tif]
