# Supplementary material for: Combined analyses of RNA-sequence and Hi-C along with GWAS loci—A novel approach to dissect keloid disorder genetic mechanism
Source: PLoS Genet. 2022 Jun 16;18(6):e1010168. doi: 10.1371/journal.pgen.1010168 (PMC9202908; doi:10.1371/journal.pgen.1010168)
Supplement: Attachment — Submitted filename: review comments and response.docx [file pgen.1010168.s018.docx]

CC: chengqi.yi@pku.edu.cn, drtirgan@gmail.com

Dear Dr Prof. Liu:

Thank you very much for submitting your Research Article entitled 'Combined analyses of RNA-sequence and Hi-C along with GWAS loci — A novel approach to dissect keloid genetic mechanism' to PLOS Genetics.

The manuscript was fully evaluated at the editorial level and by independent peer reviewers. The reviewers appreciated the attention to an important topic but identified some concerns that we ask you address in a revised manuscript

We therefore ask you to modify the manuscript according to the review recommendations. Your revisions should address the specific points made by each reviewer, and provide a detailed list of your responses to the review comments and a description of the changes you have made in the manuscript.

We hope to receive your revised manuscript within the next 30 days. If you anticipate any delay in its return, we would ask you to let us know the expected resubmission date by email to plosgenetics@plos.org.

If present, accompanying reviewer attachments should be included with this email; please notify the journal office if any appear to be missing. They will also be available for download from the link below. You can use this link to log into the system when you are ready to submit a revised version, having first consulted our [Submission Checklist](http://journals.plos.org/plosgenetics/s/submit-now#loc-submission-checklist).

While revising your submission, please upload your figure files to the [Preflight Analysis and Conversion Engine](http://pace.apexcovantage.com/) (PACE) digital diagnostic tool. PACE helps ensure that figures meet PLOS requirements. To use PACE, you must first register as a user. Then, login and navigate to the UPLOAD tab, where you will find detailed instructions on how to use the tool. If you encounter any issues or have any questions when using PACE, please email us at figures@plos.org.

Please be aware that our [data availability policy](http://journals.plos.org/plosgenetics/s/data-availability) requires that all numerical data underlying graphs or summary statistics are included with the submission, and you will need to provide this upon resubmission if not already present. In addition, we do not permit the inclusion of phrases such as "data not shown" or "unpublished results" in manuscripts. All points should be backed up by data provided with the submission.

To enhance the reproducibility of your results, we recommend that you deposit your laboratory protocols in protocols.io, where a protocol can be assigned its own identifier (DOI) such that it can be cited independently in the future. Additionally, PLOS ONE offers an option to publish peer-reviewed clinical study protocols. Read more information on sharing protocols at <https://plos.org/protocols?utm_medium=editorial-email&utm_source=authorletters&utm_campaign=protocols>

Please review your reference list to ensure that it is complete and correct. If you have cited papers that have been retracted, please include the rationale for doing so in the manuscript text, or remove these references and replace them with relevant current references. Any changes to the reference list should be mentioned in the rebuttal letter that accompanies your revised manuscript. If you need to cite a retracted article, indicate the article’s retracted status in the References list and also include a citation and full reference for the retraction notice.

PLOS has incorporated [Similarity Check](http://www.crossref.org/crosscheck.html), powered by iThenticate, into its journal-wide submission system in order to screen submitted content for originality before publication. Each PLOS journal undertakes screening on a proportion of submitted articles. You will be contacted if needed following the screening process.

To resubmit, you will need to go to the link below and 'Revise Submission' in the 'Submissions Needing Revision' folder.

Please let us know if you have any questions while making these revisions.

Yours sincerely,

Michael Tirgan, MD
Guest Editor
PLOS Genetics

Chengqi YI
Section Editor: Methods
PLOS Genetics


Dear Prof. Liu:

We thank you for submitting your manuscript to PLOS Genetics. Your Manuscript was review by reviewers. Please respond to the reviewer comments and revise and resubmit your manuscript.

Thank you
PLOS Genetics

To：

Dr. Michael Tirgan, MD
Guest Editor, PLOS Genetics
Dr. Chengqi YI
Section Editor: Methods
PLOS Genetics

Dear Dr. Tirgan and Dr. Yi:

Thank you for the opportunity to submit revised manuscript to Plos Genetics. We have carefully read all reviewers’ comments and made point-to-point responses (see below). In addition, the manuscript has been carefully revised with the revision part highlighted in **red.**

I also greatly appreciate the time and effort made by the editorial staff as well as independent reviewers on this manuscript.

Best regards,

Wei Liu, MD, PhD

Professor,

Department of Plastic and Reconstructive Surgery

Shanghai Ninth People’s Hospital, Shanghai Jiao Tong University School of Medicine

*Reviewer's Responses to Questions*

***Comments to the Authors:
Please note here if the review is uploaded as an attachment.***

*Reviewer #1: This manuscript describes an RNASeq approach combined with Hi-C analysis comparing 3 keloids with adjacent normal skin pairs and investigates the GWAS loci previously described by Nakashima et al., 2010. The authors identified several potential pathways leading to keloids and identified several genes within these pathways that showed differential expression by immunohistochemistry and performed over-expression and knock-down experiments to show effects on cell survival.
Overall, I recommend that authors revisit English grammar and sentence structure in the manuscript for better readability.*

**Response:**

Thank you for the comments, we have carefully revised the English grammar and sentence structure according to your suggestion and highlighted in **red**. Please see following response.  *L48: Please rephrase sentence as keloids don’t just appear “only” due to re-occurrence.*

**Response:**

Thank you for the comments. The original sentence means that “*keloid occurs only …. in human being*”. To avoid the confusion, the sentence is rephrased as “which only occurs **in human beings with severe reoccurrence post-therapy**.”

*L49 and L270: Do you mean the control of keloid growth and recurrence is not well understood? Please rephrase.*

**Response:**

Thank you for the comments. We intended to mean “difficult to control”, rather than not well understood. To clarify this confusion, sentences are rephrased as:

L49: “….without complete control of its growth and relapse” is changed into “…. **with difficulty to control its growth and relapse**”

L270: “…. along with uncontrolled….” is changed into “……**with difficulty to control its…..**”

*L50: replace “exon gene mutation” with “exonic gene mutations”*

**Response:**

Thank you for the comments. It has been done according to the suggestion.

*L53: replace with “by mutations in regulatory”.*

**Response:**

Thank you for the comments. It has been done according to the suggestion.

*L56: replace with “may lead to a breakthrough in the discovery of”. Do you mean further analyses are needed? Please rephrase.*

**Response:**

Yes, “given……” is the condition that mean **if** ……… To avoid the confusion, the sentence is rephrased as “**if** further large-scaled analyses **are performed** along with human keloid Hi-C data.”

*L68: recommended change to plural “gene mutations are increasingly recognized as one of the critical risk factors”.*

**Response:**

Thank you for the comments. It has been done according to the suggestion.

*L82: change to “For example, overexpression….” as this lncRNA is being used as an example and has nothing to do with keloids.*

**Response:**

Thank you for the comments. It has been done according to the suggestion.

*General comment: Were the differentially expressed genes detected in all samples uniformly or was there heterogeneity (all keloids versus all controls in your various analyses)?*

**Response:**

Thank you for the comments. Yes，they were detected in all 3-paired samples uniformly with a high efficiency. That is the reason we would like to submit the manuscript to the method section of Plos Genetics.

*This needs to be addressed upfront and also appropriately addressed in the Discussion section. For better reading, the relevant paragraphs could mention the keloid samples that were used for specific experiments [e.g., K1-K3 (Suppl. Table S1)].*

Thank you for the comments. According to the suggestion, at the very beginning of the results section, “**uniformly detected in all 3 paired samples,** **K1-K3, Table S1**” is inserted as “*The differential expressed mRNAs (DEGs), lncRNAs and miRNAs between keloids and their corresponding adjacent normal skins* ***(uniformly detected in all 3 paired samples, K1-K3, Table S1)*** *were calculated from the RNA-seq data to form a heatmap (S1 Fig)*.”

In addition, this information is also added to the discussion part as “*Due to the obvious advantages, we conducted an exploratory try of RNA-seq using a self-control model that could minimize individual variation* ***and was able to detect uniformly differentially expressed RNAs****.*” at the sixth paragraph.

*L133: hsa-miR-355-5p was down-regulated in your RNA-Seq experiments. Were the interacting genes that are listed here also DEGs in your screen? This is not clear from your sentence.*

**Response:**

Thank you for the comments. Yes, all the data presented in this manuscript are derived from the RNA-seq analysis of three-paired tissue samples. Technically, we first identified all has-miR-355-5p associated genes, and then perform intersection analysis with DEG, and finally selected the intersected genes to build up the has-miR-355-5p interaction map.

To clarify the fact, The sentence of “**The entire RNA regulation network was built on the DERNAs of three-paired tissue samples.**” is added in the beginning of result section of The RNA regulation network.

*L256: Were these biological and technical replicates?*

**Response:**

Thank you for the comments. Yes, these are biological replicates. 3 independent cell samples means 3 different cell samples.

*Was the knock-down performed with keloid samples used for RNASeq or in some of the additional keloid samples (KS1-KS9)?*

Thank you for the comments. This was done in some of the additional keloid samples, as this has been described in the method section of Patients and specimens, “Besides, nine keloid tissue samples (named KS1-KS9) were also employed for *in vitro* cell function analysis of the DERNAs.”

*Was there any heterogeneity seen in the latter samples or did all samples have a comparable knock-down result? Please expand for more clarity.*

Thank you for the comments. As usual, all knock-down results were derived from at least 3 different samples and each sample was mixed by three patient derived cells, therefore, the results were quite uniformly presented with significant statistical differences. Actually, this has been clearly indicated in the figure legend of Figure 6, “Each assay was repeated in at least 3 independent cell samples. (* P < 0.05, ** P < 0.01, *** P < 0.001)”

*L366: The fact that the samples K1-K9 are keloid-normal sample pairs needs to be described in the Methods section.*

**Response:**

Sorry for the confusion. The information regarding tissue and cell samples are provided in the method section of Patients and specimens, in which, K1-K3 were paired keloid-normal samples that were used for detecting DERNAs. Whereas other five paired tissue samples (named KT1-KT5) were used for the verification of DERNAs and corresponding proteins using IHC, WB and RT-qPCR. Besides, nine keloid tissue samples (named KS1-KS9) were also employed for *in vitro* cell function analysis of the DERNAs.

There was no K9 sample, instead, KS1-KS9 samples were used to harvest 9 keloid fibroblast samples for in vitro study, in which normal cells were not involved, therefore, KS1-KS9 were pure keloid samples without paired normal samples.

*Fig. 5 does not show or explain that these samples were used.*

**Response:**

Thank you for the comments. Based on the method description, KT1-KT5 were used for the assays shown in Figure 5. To make it clear, this information is inserted into the Fig 5 legends as “Each assay was repeated in at least three independent samples (**respectively from KT1-KT5 samples**, * P < 0.05, ** P < 0.01, *** P < 0.001)>”

*It would be clearer if transcript abundance relative to a housekeeping gene would be presented in box plots (Livak and Schmittgen, Methods, 2001; PMID: 11846609). Thus, it would also be more clear whether a message is highly expressed or at low levels.*

**Response:**

Thank you very much for the suggestion, we have carefully read the article, it is about the method and mechanism description of real-time qPCR analysis, and indeed is very helpful for understanding the mechanism.

In the practice of qPCR analysis, we used commercial and professional equipment (AppliedBiosystem) to perform the assay by strictly following the suggested protocol, therefore we believe this will be an appropriate way of presenting qPCR results, which has been published in our many previous papers. This method is also supported by many prestigious journals including PLOS genetics listed in the followings:

1. PLoS Genet. 2010 Jan;6(1):e1000805;

PLoS Genet. 2017 May 25;13(5):e1006809;

PLoS Genet. 2021 Feb 18;17(2):e1009318;

2. Immunity. 2010 Jun 25;32(6):815-27;
3. Blood. 2016 Jun 16; 127(24): 3040–3053;
4. EMBO Mol Med. 2013 Aug;5(8):1180-95;
5. Nat Genet. 2008 Jan; 40(1): 35–42;
6. Biomaterials. 2008 Dec;29(36):4792-9).

Through this way, relative expression levels for each gene of interest were normalized to that of the housekeeping gene.

*L453: WGCNA appears to be best suited to study linear relationships in a larger number of samples. You analyzed each of 3 keloids to the corresponding normal skin sample - or all samples together? Please describe your methodology and the limitations of WGCNA.*

**Response:**

WGCNA was used to construct a co-expression network and to find the modules of highly correlated genes (highly co-expressed clusters of genes). Usually, WGCNA was not applied when the samples were less than 15 ([eLife.](https://www.ncbi.nlm.nih.gov/pmc/articles/PMC5826274/) 2018; 7: e30649.). But based on the particular advantage in processing gene expression datasets of WGCNA ([Front Physiol.](https://www.ncbi.nlm.nih.gov/pmc/articles/PMC6433881/) 2019; 10: 275), it was still used in a small number of samples ([Sci Rep.](https://www.ncbi.nlm.nih.gov/pmc/articles/PMC6858342/) 2019; 9: 16910; Genomics. 2020 May;112(3):2302-2308; [Genomics Proteomics Bioinformatics.](https://www.ncbi.nlm.nih.gov/pmc/articles/PMC7801251/) 2020 Jun; 18(3): 256–270).

Moreover, genes screened from WGCNA modules in previous studies were used to process analysis like GO or KEGG ( [J Neuroinflammation.](https://www.ncbi.nlm.nih.gov/pmc/articles/PMC5391610/) 2017; 14: 84; Acta Neuropathol Commun. 2018 Mar 1;6(1):19). We used the R package “WGCNA” to identify networks of co-expressed DEGs and DElncRNAs. The p value of DEGs and DElncRNAs between keloid samples and normal skin samples was calculated and regarded as feature of each module, in order to select significant modules.

*L516: “cultivated” do you mean incubated?*

**Response:** Thank you for the comments, we changed it into “incubated”

*L513: Since TEX9 showed the opposite effect from Western blot results the methods section should describe how you did control staining for IHC. Isotype controls?*

**Response:** Thank you for the comments. Indeed, there is mismatch between IHC and WB. We did not use isotype controls for IHC, because the paired tissue serve the best self-control. Non-specific binding will stain both keloid and nearby normal skin as both were in the same tissue section and stained in the same time. Indeed, no false positive staining were found in the paired normal skin, indicating that IHC technique is adequate.

Regarding the opposite IHC and WB results, we present and respect what we got, but favor for IHC results, because:

1. IHC results match the RNA-seq data and gene expression data of keloid tissue and adjacent normal skin.
2. It also match the functional assay in vitro using keloid fibroblasts.
3. One major shortcoming of tissue based WB is that different content of extracellular matrix would affect the results. As we previously published (see below picture, Archives of Biochemistry and Biophysics 697 (2021) 108676), significant difference in matrix component can be observed. Use quantified protein content as the only indication for equal loading, one part of ECM quantity could change other part quantity. Therefore, tissue WB result could be affected by different ECM component quantity and would be less credited comparing to cell-based WB, where ECM interference can be avoided.
4. Thus, we believe IHC staining for specifically positive cells would less affected by the ECM component, likewise for gene expression analysis, all ECM proteins were excluded and would not interfere the results.

Archives of Biochemistry and Biophysics 697 (2021) 108676)

*Fig.1: This is an interesting presentation of a summary but may be easier to read as a flow chart.*

**Response:**

Thank you for the comments. Indeed, it appears to be easier to read as a flow chart. We originally had prepared a flow chat, but seemed to be difficult to understand due to the complicated strategies. Therefore, we specifically designed a 3D figure to provide a clear visual image for easy understanding. We would appreciate if the original Figure 1 can be maintained in the revised manuscript.

*Fig. 2: Please spell out WGCNA in the figure legend.*

**Response:**

Thank you for the comments. “**weighted gene co-expression network analysis**” is added into Fig. 2 legend.

*What do colors in 2c stand for? Needs to be mentioned somewhere.*

**Response:**

Thank you for the comments. Seven gene co-expression modules were detected by WGCNA and three modules (blue, red, and turquoise) showed to be significant difference with p<0.05, which meant that molecules in these three modules were most significantly differentially expressed between keloid samples and healthy skin samples (Fig 2C and S2 Fig).

To help understand, in the Fig. 2 legend, “the paired DEG and DElncRNA were divided into 7 modules by WGCNA” was replaced by “**Seven gene co-expression modules represented by 7 different colors were detected by WGCNA from the paired DEG and DElncRNA”.**

*Fig 3D: The Western bands are presumably not from a single blot, yet only one housekeeping band is shown. Can you quantify the keloid/normal scar bands to their respective housekeeping gene?*

**Response:**

Thank you for the comments. Yes, the bands shown in Fig. 3D were from 2 blots that run the gels at the same time. In another word, 12 tissue samples were loaded onto two gel, each blot contained 12 lanes for 6 paired samples. After membrane transfer, two membranes were respectively cut into 12 small blots, i.e. one small blot for 1 paired samples, and then each small blot was incubated with one antibody. Therefore, no extra β-actin was available. Since the samples were equally loaded at the same time, we thus believe one housekeeping band would be suitable for all tested bands.

This WB presentation style has been uniformly employed in most prestigious journal, such as:

1. Blood. 2016 Apr 7; 127(14): 1780–1789;
2. EMBO Mol Med. 2018 Nov;10(11):e9408.
3. Vet Microbiol. 2011 Apr 21;149(1-2):147-56;
4. Eur Heart J. 2014 Apr;35(14):911-9;).

In addition, it is certainly possible to quantify protein bands to their respective house keep bands. As requested by the journal, we will submit numeral data along with the WB data.

Quantify protein bands of Figure 3D and Figure 5C.

|  | NS | KS |
| --- | --- | --- |
| LAMA3 | 15896 | 28569 |
| LAMA2 | 18283 | 6778 |
| LAMA1 | 23545 | 13379 |
| ERBB3 | 18281 | 3825 |
| ERBB1 | 20288 | 12776 |
| Slit2 | 24159 | 7286 |
| IGF1R | 11472 | 22122 |
| ICAM1 | 22528 | 17167 |
| PPARy | 17197 | 9625 |
| L-selectin | 20683 | 20372 |
| Areg | 17811 | 6948 |
| β-actin | 36476 | 37635 |

|  | NS | KS |
| --- | --- | --- |
| NEDD4 | 5184 | 32482 |
| HHIPL2 | 2101 | 6148 |
| PYGO1 | 3158 | 8126 |
| TEX9 | 5018 | 10185 |
| RAB27A | 7594 | 9187 |
| β-actin | 30158 | 29483 |

*Fig 5D: It would be clearer if transcript abundance relative to a housekeeping gene would be presented in box plots. Thus, it would also be more clear whether a message is highly expressed or at low levels.*

**Response:**

Thank you for the comments. It has been addressed above and your understanding will be greatly appreciated.

*Reviewer #2: This study focuses on keloid disorder, a group of fibroproliferative tumors, keloids being the prototype. These studies are welcome in the context of the notion that there are no effective or specific treatments for this disorder. The study is a methodological tour-de-force combining several state of the art technologies, including GWAS, RNA-seq and Hi-C, analyses, and the data are subjected to extensive bioinformatics, which has generated a large number of data which have been meticulously catalogue in this manuscript.
There were a number of issues at that should be considered by the authors for improvement of the study.*
**Response:**

Thank you for the comments, which is very valuable for improving this and future manuscripts. The followings are the detailed response.

*1. The study focuses on keloids but only three individual keloid samples, together with the adjacent normal skin, were analyzed.*

**Response:**

Thank you for the comments, this study tried to prove the efficacy, efficiency and high reliability in the prediction of potential mechanism by further verification with other 5 paired tissue and 9 keloid samples *ex vivo* and *in vitro*. It is also widely applied for various disease investigation as supported by the references [4-7].

*The author's description of the keloids is somewhat incompletes in that the authors do not make a clear distinction between keloids and hypertrophic scars. In fact, the description in the first paragraph of the Introduction is more applicable to hypertrophic scars.*

**Response:**

Thank you for this important comment and we appreciate it very much. Usually, invasion into normal skin and lack of spontaneous regression are the characters to distinguish it from hypertrophic scar. To make it clear, the sentence is rephrased as “*Keloid is considered as a benign skin tumour that exhibits many cancer-like characteristics such as uncontrollable and invasive growth* ***with*** *extremely high recurrence rate.* ***Unlike hypertrophic scar, keloids are*** *lack of spontaneous regression* *and* ***able to cross the original wound boundary.***”

*Furthermore, there is no histological documentation of the type of keloid tissue that is being analyzed.*

**Response:**

Thank you for the comments and we fully agree with and appreciate your points. Keloids are generally divided into two types, tumor-like keloid and inflammatory keloids. The second type some time is difficult to distinguish from hypertrophic scar. But the tumor-like keloids are unique and not resemble hypertrophic scar at all.

From the very beginning, we introduce the cancer-like characters of keloids, and this study involved only tumor-like keloid type. As illustrated in Figure 2A, all three selected cases exhibited nodular or cauliflower growth types, that never happens to hypertrophic scar. In addition, all three cases were originated from spontaneous formation which never happens to hypertrophic scar as well. Therefore, we don’t believe that we need to show histology image to prove that they were keloids but not hypertrophic scars.

*The importance of this point is emphasized by the fact that keloid lesions can be highly heterogeneous, some parts of the lesions being highly cellular with a large number of inflammatory cells, while other parts are characterized by dense fiber meshwork of extracellular matrix of connective tissue, mostly collagen, with a few myofibroblastic cells embedded into it. Thus, it is not clear how representative their findings are in the spectrum of keloid pathology.*

**Response:**

Thank you for the comments. We certainly agree with your view point that keloids are heterogeneous and that is the reason why we only investigate tumor-like type keloids. Regarding different regions of keloids, they may exhibit different pathological statuses, we also agree with that. In this study, all keloid tissues were harvested from the peripheral active part of keloids. To make it clear, we highlight **“Then three pathological specimens from the outer zone of keloid lesions as well as three adjacent normal skins were collected during keloid lesion resection surgery.”** In the section of **Patients and specimens**  *2. While the genetic contribution to keloid development is well established through population studies, the authors state that “genetic contribution is well expected due to the presence of autosomal dominant inheritance.” In fact, autosomal dominant inheritance has been documented only in a very few families. In the present manuscript, there is no reference to family history of keloids in the three patients studied.*

**Response:**

Thank you for the comments and we fully agree with your points. Rather than authors’ concept, the senescence “*genetic contribution is well expected due to the presence of autosomal dominant inheritance*.” is paved for the reasons why quite some GWAS studies were performed. To clarify the concept, the sentence is rephrased as “*Genetic contribution is well expected due to the presence of autosomal dominant inheritance* ***and various genetic mutations in keloid lesions.***” *3. Additional studies were performed using cultured keloid fibroblasts Isolated from apparently inflammatory outer zone of the lesions. The pitfall of these experiments relate to the culture conditions of the fibroblasts. First, the cells were cultured on plastic culture dishes, but it has been well documented that the culture of keloid fibroblasts under such standard fibroblast culture conditions results in loss of the collagen overproducing phenotype, and the keloid fibroblasts in culture do not recapitulate the characteristics of cells in keloid lesions in situ. Consequently, it is very unclear if the changes observed in the molecular studies are really representative of the characteristics of these cells when part of the keloid lesions.*

**Response:**

Thank you for the comments. We generally agree with your view points. The differences in cell behaviors between *in vitro* and *in vivo* environment are uniformly presented in all biological studies including cancer, development biology study, wound healing, cell metabolism, etc. The difference does not exclude the necessity of *in vitro* cellular study in order to understand the mechanism or verify the functions of important signaling pathways.

Likewise, *in vitro* investigation of keloid cell behavior has been well established to understand keloid mechanism and to verify *in vivo* finding in many published literatures. It is generally recognized that keloid cells remained largely able to maintain its pathological phenotype as long as the first 3 passage cells are used for the study as supported by numerous keloid *in vitro* studies. In addition, the *in vitro* part of this study mostly used the passage 1 cells in order to better correlate in vivo findings.

Furthermore, almost all *in vitro* studies of keloids used the peripheral-zone (active stage) to represent keloid *in vivo* status, and this was what we have done. Interestingly, the cellular functional assays unanimously supported the functions of cell cycle arresting and induced apoptosis, when biomarkers were over-expressed or knockdowned to oppose *in vivo* expression profiles of these molecules. This unanimous functional verification could not happen randomly, rather it proved the right selection of these biomarkers.

*4. Another deficiency of the culture conditions is the apparent lack of ascorbic acid (vitamin C) which is an obligatory requirement for the synthesis of not only collagens but also a number of other proteins, and the corresponding mRNA transcript levels have been shown to be altered by addition of vitamin C to the tissue culture medium.*

**Response:**

Thank you for the comments. It is true that adding vitamin C was essential for increasing collagen synthesis, but surely won’t affect cell cycle and apoptosis status. This study focused on cells’ tumor-like behavior change by mimicking cancer cell biology studies that usually don’t add Vit C in their study as supported by the following supporting references *(Int J Mol Sci. 2020 Nov; 21(21): 8050; Cell Death Dis. 2021 Jan; 12(1): 40; Redox Biol. 2021 Jan; 38: 101815).*

Nevertheless, your point is well taken and VitC was actually added in our other keloid studies in which abnormal matrix production was the focus of the investigation.

*5. The bioinformatics of the data derived from the molecular analyse, I.e., GWAS, RNA-seq and Hi-C, has resulted in a large number of data that are faithfully detailed in the manuscript. It is apparent, however, that many of the pathways identified to be altered in keloid samples vs. control skin may have very little, if anything, to do with the pathomechanisms of keloids. Thus, while technically for the most part correct, these studies have provided information which is very difficult to correlate to the development and growth of keloids, with little therapeutic implications for this, currently intractable disorder. As indicated by the authors, this is really an exploratory study with a limited number of samples and an increase in sample size and advancements in sequencing technologies are required to dissect the pathological changes associated with keloids.*

**Response:**

Thank you for the comments. We well respect your view points and they might be true from the fact that no scientific investigation could fully recapture the true *in vivo* mechanistic change. However, we do not necessarily agree with the points that “these data are likely useless and nothing to do with true keloid pathological development” based on the following reasons:

1. The strategies and the technologies are well established and employed in most of cancer biology studies, which generate enormous bioinformatics information proved to be valuable for understanding cancer mechanism and finding therapeutic targets. We did the same thing by investigating keloid using the same strategy.
2. The identified important pathways have been partially reported by the literature, such as Wnt signalling pathway and PPARγ signalling pathway[40], ECM‑receptor interaction pathway [41]. Whereas some novel findings are also supported by our unpublished data. For example, abnormality of cytochrome P450 in this study was found in our keloid single cell sequence analysis. In addition, estrogen signalling pathway can be verified by the clinical phenomenon that keloids rapidly grow during pregnancy stage. The biomarkers’ functions were also verified by the *in vitro* studies. The most DEmiRNA, i.e. miR-335-5p, has been documented in the literature for cancer development, it might have important function for keloid development given that keloids represent benign skin tumor.
3. The advantage of this manuscript is to provide huge amounts of bioinformatics data, which will help for future investigations with valuable and available information. While it seems “little to do with keloid development,” this is because no possibility to functionally validate all data in a single paper. In addition, with the advancement of bioinformatics and genetic technologies as well as the accumulation of biological data, these seemingly non-related information pieces could be Jigsaw puzzled into a meaningful keloid mechanism map.
4. Although large sample size and keloid hi-C data are preferred in future study, the current study with a small sample size already demonstrated high efficacy, efficiency and high reliability, indicating a right methodology strategy for future keloid mechanism study.

*Reviewer#3*

*The authors have undertaken a very important step in better understanding the genetics of keloid disorder. This work deserves to be published; however, this manuscript can be much improved prior to being published.  Please consider the following:*

**Response:**

Thank you very much for the supportive comments and we provide detailed response according to your suggestions in the followings.

1- *The title of the manuscript - Keloid-specific gene expression profiling – will easily attract the readers who are familiar with keloid disorder and are interested in this topic, therefore there is no need to provide an exhaustive review of what a scar is, or what a keloid is. Furthermore, there is no general agreement on the definitions that are provided for scar, hypertrophic scar, etc.*

**Response:**

Thank you for the comments. Indeed, the suggested title would be more attractive. However, since we submit the manuscript to method section of Plos Genetics, we thus would like to maintain the original title as it faithfully describes the main points of this study.

*I would encourage the authors to remove much of introduction and start their abstract from where they state: “In an effort to find out keloid-specific genes---” and the introduction from where they state: “To date, there have been five reports---".  Those interested in this publication are very familiar with the underlying disorder.*

**Response:**

Thank you for the comments, we made changes where necessarily.

*2- Authors refer to Keloids as Scars. Keloids are not scars. The authors have somewhat alluded to this in the context of their manuscript. The authors should more precisely define different pathologies, and perhaps consider the following:*

*Consider using the term “Keloid Disorder” to refer to the underlying genetic disorder that keloid patients have.*

*Consider using the term “Keloid Lesion” to refer to the actual lesions.*

*Consider using the term “Keloid Tissue” to refer to the tissue of a keloid lesion.*

*Consider using the term “Keloid Patient” to refer to the patient who suffers from keloid disorder.*

*Avoid using the term “Keloid” alone, as using this term alone leaved the reader to decide is the author is talking about the Keloid Disorder or the Keloid Lesions.*

*Avoid using “exaggerated scars such as hypertrophic scars and keloids” Just state hypertrophic scars and keloid lesions.*

**Response:**

Thank you for the comments, we made changes where keloids are properly replaced with keloid disorder, keloid lesions and keloid patients.

*3- The core of the introduction starts where the authors state: “To date, there have been five reports comparing gene expression patterns in keloid and related scar lesions—”*

*Authors should consider using a phrase such as: To our knowledge, there have been five reports comparing gene expression patterns in keloid tissue.*

*Also, “related scar lesions” is vague. What do you mean by related scar lesions? In the same patient, in other patients.*

**Response:**

Thank you for the comments, we have tried to perform necessary changes.

*4 - In the introduction section, the authors state: “In this study, we aimed to compare the overall gene expression patterns of reversible and irreversible scars and provide insight into the molecular and cellular mechanisms behind these lesions.”*

*Please revise the phrase “irreversible scars such as keloids and reversible scars.” This is way too confusing. Tell us what you exactly did.*

**Response:**

Thank you for the comments, and we fully agree with your comments.

*5- In the introduction section, the authors state: “abnormal fibroproliferative scar lesions,”*

*The authors use several different terms, and it is unclear what they mean by these. For instance, in here, do you mean Keloid Lesions?*

**Response:**

Thank you for the comments, and we fully agree with your comments. We have used proper terms of disorder, lesion and tissue as above suggested. Therefore, keloid disorder is used to describe the disease in the revised manuscript.

*6- In the result section, the authors stated that” To reveal the gene expression characteristics of keloids and related lesions, we collected 4 keloid tissues, 5 hypertrophic scars, and 3 normotrophic scars.”*

*Were these tissue samples taken from ONE patient? Or from 12 different patients?  Authors need to better describe the origin of the tissues that they analyzed. Did 4 keloids come from 4 different patients or from one keloid patient? Same as the other tissues.*

**Response:**

Thank you for the comments and sorry for the possible confusion.

In this revised manuscript, in the method section of Keloid patients and samples, you will find that three paired keloid lesion-normal skin samples (named as K1-K3) were used for RNA-seq and bioinformatics analysis. Another 5 paired keloid lesion-normal skin samples (named as KT1-KT5) were employed for verification assays in HIS, WB and RT-qPCR. Another 9 keloid tissue samples (named as KS1-K30) were employed for keloid fibroblast extraction for in vitro study. No hypertrophic scar and normotrophic scars are included.
